# Expressed breast milk as 'connection' and its influence on the construction of 'motherhood' for mothers of preterm infants: a qualitative study

**DOI:** 10.1186/1746-4358-3-30

**Published:** 2008-12-17

**Authors:** Linda Sweet

**Affiliations:** 1 Flinders University Rural Clinical School, Flinders University, Adelaide, SA, Australia

## Abstract

**Background:**

Breast milk is considered the optimal nutrition for all newborn infants. While there is high initiation of lactation among mothers of preterm infants in Australia, there is a rapid decline of continued lactation. Furthermore, there is an inverse relationship between infant gestation and duration of lactation. To better understand the breastfeeding experience of parents of very low birth weight (VLBW) preterm infants an interpretive phenomenological study was conducted.

**Methods:**

This longitudinal study was conducted using an interpretive phenomenological approach. Data were collected from 17 parents through 45 individual interviews with both mothers and fathers, from birth to 12 months of age. This data was then transcribed verbatim and analysed using thematic analysis.

**Results:**

The analysis identified six primary themes: the intention to breastfeed naturally; breast milk as connection; the maternal role of breast milk producer; breast milk as the object of attention; breastfeeding and parenting the hospitalised baby and the demise of breastfeeding. This paper reports on the theme of 'breast milk as connection'.

Providing expressed breast milk offered one way the mothers could be physiologically and emotionally connected to their preterm infant while they were in the constant care of hospital staff. Indeed, breast milk was considered the only way the new mother could connect her body (or part there of) to her preterm baby in hospital. This sense of connection however, comes at a cost. On the one hand, the breast milk offers a feeling of connection to the baby, but, on the other, this connection comes only after disconnection of the mother and baby and – through breast expression – mother and her milk. This ability of breast milk to connect mother and baby makes the expressed breast milk highly valued, and places unexpected pressure on the mother to produce milk as integral to her sense of motherhood.

**Conclusion:**

The findings of this study have implications for healthcare practice. It is evident that the association of breastfeeding success with mothering success only jeopardises some families' self-esteem and sense of parenting ability. These findings suggest it would be beneficial to find alternate ways to connect preterm infants and their parents in the preterm nursery environment, and find more positive ways to support breastfeeding.

## Background

Breastfeeding is widely accepted and advocated as the best source of nutrition for newborn infants. While there is a high initiation rate of lactation among mothers in Australia, it is known that for preterm infants there is a rapid decline during the weeks and months following the birth, at a rate greater than their term infant counterparts [1,2]. The breastfeeding experiences for these families are inherently different to those of healthy term infants, as the newborn preterm infant is too weak and immature to feed directly from the breast and long-term breast expression becomes necessary.

Preterm birth results in infants requiring long-term hospitalisation and family separation. The experience of having a child admitted to a Neonatal Intensive Care Unit (NICU) undoubtedly creates a stressful situation for the parents [3-7]. Fenwick conducted a study of parenting in Australian neonatal nurseries and concluded that mothers struggled through intense emotional, cognitive and 'worry work' in an attempt to become 'real' mothers to their hospitalised infants [8]. The new mother must commence lactation during this time of intense turmoil. Mother-infant separation has been recognised as a barrier to successful breastfeeding [9,10]. All breastfeeding mothers have concerns and problems [11]. However, when it involves a preterm infant, the concern in the current literature frequently shifts to the baby. The voices of parents at this time have been neglected in the professional literature. There are many studies on processes to initiate and maintain sufficient lactation following the birth of a preterm baby, but few have specifically studied parents' issues of breastfeeding preterm infants.

Breastfeeding is the norm in contemporary Australian society. Recent Australian research has demonstrated an association between breastfeeding and maternal identity. Cooke argues that breastfeeding is instinctive – an emotional activity – and is associated with maternal identity and self-esteem [12]. Schmied's participant women espoused the current socio-political discourse in favour of breastfeeding at all costs and constructed breastfeeding as something they wanted (and needed) to achieve for their motherhood identity [13]. Indeed much of the research investigating women's perceptions of breastfeeding over the past 20 years has some striking commonalities. It is frequently reported that women demonstrate a strong conviction that breastfeeding is best for babies, while breastfeeding is considered synonymous with being a good mother and a woman's identity or sense of self as a mother [11,12,14-21]. These studies, however, centre on mothers of healthy term infants.

Therefore, there is an absence of research investigating the parents' voices and the ways in which they experience breastfeeding for a preterm infant. Furthermore, it is unknown how mothers construct expressing for their hospitalised preterm infants in light of the literature which suggests breastfeeding is synonymous with a mother's sense of motherhood. It is this lack of experiential knowledge in the professional literature that has led to this research study.

Phenomenology is a philosophical view of the world in which there is an inseparable connectedness of human beings to the world. Consequently, the world cannot be separated into subject and object; rather, the world can only be described as experienced by human beings. Furthermore, human beings make meaning from lived experiences. It is considering this consciousness, of being in the world that guides phenomenological interpretation, and this study. As van Manen explains:

*"the world is given to us and actively constitutes by us: reflecting on it phenomenologically, we may be presented with possibilities of individual and collective self-understanding and thoughtful praxis" *[[22]* p. xi*]

The participants in this study anticipated 'normal' breastfeeding with expectations of what it ought to be. This study explores the lived reality of breastfeeding for a preterm infant from people living the experience.

## Method

This study was conducted to answer the research question: what is the lived experience of parents breastfeeding very low birth weight preterm infants? To enable exploration of the experience from the perspectives of both mothers and fathers, an interpretive phenomenological approach was used. Interpretive phenomenology is a qualitative research approach that systematically investigates people's lives, experiences, understandings and perceptions of what it means to be human [23]. Interpretive phenomenology is a science which is interested in the study of people, of what it is to be human, and offers an advance of our knowledge by increasing our understanding of lived experience. Interpretive phenomenology enables researchers to uncover and interpret the many ways in which people articulate and make sense of their experiences related to health and illness. Thus, phenomenological insight goes beyond merely an adequate technical knowledge [24] – it improves our understanding of the meaning of an experience and is therefore a suitable method for this research question. Phenomenology as a research method is useful to guide pathic understanding of human life, which, in turn, is useful to guide empathetic nursing action [25]. Therefore people's own individual experiences are worthy forms of data to be studied. To conduct phenomenological research is to question the way we experience the world.

Interpretive phenomenology is a philosophical tradition of reading, reflection and rewriting. It is therefore important to recognise (or remind ourselves) that the goal of interpretive phenomenology is to understand 'Being' [23]. When using interpretive phenomenology, the researcher seeks to give greater access and understanding of the text in its own terms, thereby allowing the reader to notice meanings and qualitative distinctions within the text for themselves as well as from the dialogue provided [26].

This study was conducted in an Australian metropolitan hospital during 1999 as a supervised doctoral research study. Partial results of this study have been published elsewhere [27,28], and this paper presents data not yet published. Ethical approval was gained from both the Hospital and University ethics committees and all local and national research guidelines pertaining to informed consent, participant confidentiality and anonymity were adhered to.

The sample consisted of 10 mothers and 7 fathers who intended their preterm very low birth weight (VLBW) baby to be breastfeed. All potential participants were identified from the admissions register in the NICU and parents were approached by the researcher and recruited to the study in the first week after the preterm birth. Parents were excluded from selection if they did not speak English; if their infant had a congenital abnormality likely to affect feeding; or if their infant was considered gravely ill by the attending neonatologist. The researcher had no clinical role in the hospital and therefore was not associated with the care of the mothers or babies.

Data was generated through 45 semi-structured individual interviews. Three interviews were scheduled with each participant; at 2–3 weeks, 8–10 weeks and 12 months post birth. Some parents chose not to complete all three interviews and two families were not contactable for the final interview. These interviews were done in private interview rooms at the hospital, the parents' own home or over the telephone as chosen by the participants. No interview schedule was applied, but rather a spider map of keywords was used to bring up new ideas and areas for further discussion. Participants were encouraged to share their own stories in their own way, proceeding on their own terms while describing aspects important to their own experience. The questions posed during the interviews revolved around the topics of conversation as directed by the participants. To enable spontaneity of discussion, interviews were audio-tape recorded, then transcribed verbatim. Transcripts were analysed using thematic analysis outlined by Benner [23] which sought to highlight and explore the narrated experiences, perceptions, salient events, discursive patterns and changes over time articulated by the participants. Data analysis and management were supported with the use of N-Vivo – a computer program designed for qualitative data.

Interpretive phenomenology acknowledges that research is necessarily a researcher's interpretation of participants' articulated experiences, and this paper therefore presents the authors interpretation from the data generated from the 17 participants. Qualitative research is context dependent and centred in the time, place and persons from which it has been derived. Furthermore, this study is based upon a group of white, Anglo-Australian heterosexual men and women, and thus interpretations presented cannot be automatically generalised to the broader parent population. However, it is the intent of the author to provide a justified interpretation for the reader, showing similarities and differences evident within the data.

## Results

Table 1 outlines the characteristics of the ten participating families. Given that this paper presents the mothers' construction of motherhood, the data from the 10 mothers will predominate.

**Table 1:** Characteristics of participant families

**Participant reference**	**Gender**	**Number of interviews**	**Assisted reproduct-ion**	**Single or multiple birth**	**Previous children (n)**	**Distance of usual residence to hospital (kilometres)**
1A Sue	Female	3	yes	twins	0	< 10
1B Peter	Male	3	yes	twins	0	< 10
2A Julie	Female	3	no	twins	1	10 – 200
2B Colin	Male	3	no	twins	1	10 – 200
3A Bev	Female	3	yes	twins	0	> 200
3B Brian	Male	3	yes	twins	0	> 200
4A Lisa	Female	3	yes	single	0	< 10
4B Paul	Male	3	yes	single	0	< 10
5A Chris	Female	3	no	single	1	Interstate
5B John	Male	3	no	single	1	Interstate
6A Sharon	Female	1	no	single	3	10 – 200
7A Alison	Female	3	yes	single	0	> 200
7B Dennis	Male	3	yes	single	one	> 200
8A Fiona	Female	2	no	single	nil	10 – 200
9A Helen	Female	3	no	twins	three	10 – 200
9B Wayne	Male	2	no	twins	three	10 – 200
10A Nicole	Female	1	no	single	nil	< 10

Following the preterm birth, all of the participant parents displayed overwhelming care and concern for the wellbeing of their baby. Infant survival was of paramount importance and any efforts to breastfeed were overlooked in the early hours and days of the infant's life. The parents were keen to do whatever was in the best interests of their baby. The parents were able to visit their baby but were very limited in the tangible things that they could do for their baby. Once commenced, preterm breastfeeding and provision of milk for their baby was the primary task assigned to mothers at this time. Breast expression gave all of the participant mothers a feeling of contribution. The mothers in this study expressed a level of satisfaction through 'breastfeeding': of providing something beneficial for their baby that the hospital staff could not provide. Fiona said providing breast milk made her feel like a mother when she felt she had no other mothering role:

"I like, I definitely like the idea of her having my milk and it's like I'm giving her something to grow and it's definitely, I like, I do like that idea. That she needs me to grow, it makes me feel, you know, a little bit more like a mother than just, you know, an outsider just looking at her."

For Fiona, her breast milk was something only she could provide that she felt was integral to her baby's need to grow and be healthy. All participants made reference to 'my milk' in a way that highlights its value and places it in high regard. This sense of being the exclusive provider of something her baby required placed a special importance on the mother, her breastfeeding role and particularly on her milk. Fiona did not speak of at-breast feeding being important at this time; it was simply the breast milk that the baby needed and providing this gave her a connection to the baby. Providing breast milk was the only mothering role Fiona felt able to do; otherwise all she would be able to do would be to sit and observe her baby while the hands-on tasks were done by the nurses. The theme of breast milk as connection will be presented through the following sub-themes followed by a discussion and integration with the current literature.

### 1. Breastfeeding is initially not a priority

For all participant mothers, there was a delay in their initiation of breast expression following birth. No mother expressed her breast milk on the day of the preterm birth. Breast expression was commenced by a few mothers the day after the birth; however, most did not express their breasts regularly until 2–3 days after the preterm birth. Mothers spent most of their time watching their baby and recovering from the birth themselves in those first few days. This delayed initiation of breast expression is incongruous with current research recommendations that suggest the earlier breast expression is commenced the better the lactational outcome [29-31]. However, in order to understand this experience it is valuable to look at the context and circumstances surrounding the preterm birth and the initiation of expressing, and not just the time that expressing was commenced.

The first few days of life for the baby were significant in affecting the commencement of breast expression. Participants spoke of many factors that negatively affected the initiation of their breast expression. Alison found she spent all of her day by the baby's bedside and just forgot to express:

"My main concern was him, you know, even though they say express or whatever, I was never in my room, I was always downstairs, ... and all I'm concerned about is him medically, how he is, you know, not my milk supply, you know."

Julie did not express until three days after the birth, and she attributes her delay to express to the ill health of her twin babies. Julie felt that the staff did not encourage early initiation of expressing for her at the time, as her twins were considered critically ill. Once started, expressing her breast milk did give Julie hope that all was going to be all right:

"And I wasn't bothered the fact that I was starting to do it, I was quite happy to do it, because that sort of gave me hope that they were going to be alright, that mum's milk's going to help them."

Being encouraged to start breast expressing, even days after the delivery, was a welcomed sign of hope for Julie. The sense that her milk would help her babies get better was powerful for Julie and very important for her to provide.

Sharon commenced breast expression a few days after the birth. The reasons for the delay were not clear to Sharon and she would have welcomed the ability to provide breast milk sooner. She spoke of breastfeeding as a way to feel like a mother as opposed to someone who had simply had an operation:

"They let me go for the first two [days] ... but I didn't feel as though I'd had a child, though, because those first two days I used to sit around doing nothing, I had no child in the room with me ... so I think it's probably better off in a way for the mothers to sort of start doing it [expressing] the next day. But, ... if I'd gotten on to it [expressing] straightaway it would have made me feel like a mother and not just someone that had an operation in the hospital. I certainly didn't feel like a mother the first two days."

Once expressing was commenced, Sharon found that providing her breast milk was one way she could be mother to her baby while she was in need of intensive care support. Early breast expression would have enabled these mothers to actively mother in the absence of their babies by their sides.

Mothers felt that breastfeeding for a preterm infant was not treated as a priority by many of the hospital staff in the first few days after birth. The midwives on the postnatal ward did not put any pressure on the mothers to express their breast milk. Alison felt that the postnatal midwives forgot about the mothers of preterm infants. She found the midwives on the postnatal ward concentrated on those mothers who had their babies with them, while the midwives in the NICU concentrated on the sick or preterm baby's needs. This lack of recognition as breastfeeding mothers further compounded a sense of isolation. She explains:

"... because I didn't have Joel with me, you don't have the nurses and all the people kind of giving you all this information, they're dealing, you know, with the breastfeeding issue [s] with the women whose babies are right next to them. Me without having my baby, I didn't have, like, the nurses coming in and having chats to me, you know, they just showed me the machine, what I had to do and that was the end of it, you know. ... But, yeah, the whole breastfeeding thing wasn't a priority or a major issue because my baby wasn't with me."

Alison felt that there was no staff member concentrating on the needs of the mothers whose babies were in the nurseries. This sense of isolation was from other mothers who did have their babies, from staff who cared for them, from their own baby and from an active mothering role.

The production of breast milk is commenced for nurturing a live newborn. To commence breast expression for a sick or preterm infant is assuming the infant's survival. The low importance placed on early breast expression by staff could be related to the potential demise of the sick infant; the absence of the baby with the mother in the postnatal ward; or possibly to the lack of need for breast milk to feed the baby with in those early days. Whatever the reason, the lack of encouragement from some of the hospital staff sent a powerful message to these new parents – that the baby's immediate needs were the focus of attention and that breast milk was not valued at this time.

By the end of the first week after birth, mothers were being encouraged to express their breast milk. Helen spoke of this 'step' of the preterm breastfeeding experience:

"... And I know why they do it, because they want you to focus on that more, because that's the next step, the milk supply. Like you've got over the section ... and now I have to really focus in on the milk."

Helen believed that her traumatic birth and her infants' first critical few days of life were the factors that affected the commencement of her lactation. Helen suggested that once she had coped with these early crises the expressing was introduced by the staff as the next important task.

### 2. Being a 'good' parent

Throughout the entire preterm birth experience there was a sense of all participant parents being committed to their newborn baby just as any new parent is to their newborn infant. The baby's needs were always the first priority for these families and, given the prematurity of the baby, putting the baby first was seen as even more important. Parents were prepared to do what ever the hospital staff suggested as potentially beneficial for the preterm newborn. With the newborn infant in hospital, parents constantly strove to achieve the ideal of the 'good' parent. Breastfeeding, being the feeding of choice for these parents and the hospital staff alike, was seen as something done by a 'good' parent.

Parents continually spoke of breastfeeding as being best for their baby, of offering great benefits to their preterm infant. The 'best for baby' focus was present throughout the entire breastfeeding experience from intention to cessation. Because of the strong focus on the baby, the ways in which parents spoke of the reasons or benefits for their breastfeeding were limited. Julie said:

"So everything you're doing is for your girls, I think. And I suppose, I suppose you do it for yourself too. But I don't think, I personally don't benefit anything from it, if you can understand that. I don't see it for my benefit at all, it's for my girls' benefit."

By demonstrating only child-focused benefits Julie denied her own agency. This practice of only presenting child-focused discussions and decision-making was common among participants as one way to demonstrate being a good parent – a good parent always puts the child first. To make a decision against what is best for the baby would be extremely difficult and with breast milk the known best milk for her babies, any such decision would be against the notion of being a 'good parent'.

All parents spoke of breastfeeding being synonymous with 'good' parenting. This finding is also present in the works of, for example, MacLean [15], Maushart [19], Schmied [13] and Fenwick [8]. This is not surprising given that the dominant discourses on infant feeding insist that 'breast is best' for the infant's physical and emotional wellbeing [32]. Furthermore, "'good mothers" are expected to place their infant's needs above their own and deal cheerfully and patiently with the loss of sleep and time for oneself and other privations that caring for a baby entails' [[32], p. 1012]. The participants spoke of being willing and able to do whatever this preterm breastfeeding required in order to perform as a good parent.

### 3. Breastfeeding as a marker of 'good' motherhood

While participants could see that they had to express in order to establish and maintain lactation, and to provide milk to their preterm baby who was too weak initially to feed at the breast, they also felt it had a morality for them. Many of the participants spoke of breastfeeding as an integral part of being a mother; it was a mother's job – an inherited role of having a baby. Julie expressed a strong belief that breastfeeding was a mother's job:

"Because you're the only one that can do it, no one else can do it for you."

"Um, I don't know, it might be just a mother thing, I mean you hear these women that just don't even bother trying because, you know, I can't stand that. I suppose it's a natural thing, that's what we've got boobs for, isn't it?"

Julie felt an obligation to breastfeed because it is a job of motherhood; physically and morally it was her gendered role. Bev felt that breastfeeding was integral to motherhood and something she just had to do as a mother:

"Like I said, I've said I'd feel terrible, I'd feel guilty, so, yeah, I would feel guilty if they were on formula and I knew that I, you know, I could, you know ... if you tried a little bit more, or whatever, to keep it going, because I really want to keep them on breast milk if I can."

For Bev, good mothers breastfed, and she felt a moral imperative to work hard at her lactation for her twins. Succumbing to formula was interpreted by Bev as failing at this one aspect of motherhood. In order to be a good mother there was no alternative for her other than to persevere with breastfeeding, despite the difficulties involved following the preterm birth. She could not stand back and watch the hospital staff care for her babies if she were not doing what she could to help the babies also, which included breastfeeding.

Breastfeeding was seen by the participants as something a mother needed to do for her baby. It was seen as something done by a good mother and therefore was viewed as a marker of good motherhood. This evaluation of 'goodness' of motherhood gave the women a sense of moral imperativeness to succeed at their breastfeeding despite the difficulties that arose. In the context of the hospitalised preterm infant, breast milk expression was their only tangible mothering role; thus they experienced significant pressure to succeed at this one role that they could perform. These mothers felt that their status as the mother and as a good mother was related to their ability to provide sufficient milk.

Sue was successful in providing sufficient milk but feels that if she had not been able to then she certainly would have felt guilty and inadequate:

"I probably would have felt quite disappointed, I guess, if I wasn't able to do it and maybe I would have felt as though I was letting them down a bit or maybe I was inadequate or something like that."

Mothers who were unable to meet their infants' needs found this a negative impact on their performance. Chris expressed her disappointment when her breast milk volumes did not meet the demand:

"Yeah, inadequate a bit. Like you weren't doing what you were supposed to be doing, or getting, that you were a bit of a failure."

Chris struggled to keep up her milk supply throughout the baby's hospital stay, and her sense of inadequacy never went away. Alison also had trouble supplying sufficient milk for her baby. Alison spoke of breastfeeding as being her role and responsibility, and spoke of feelings of guilt regarding her inadequate milk volume:

"... It is my responsibility, because I want the best for him you know. And my milk is the best for him, there's no two ways about that, you know, and like I said ... I think every time I have little milk I feel guilty."

Some mothers saw breastfeeding duration as another marker of good motherhood. Julie was concerned that after having decided to breastfeed, changing her mind and giving up would be inferred as failure or as not being a good mother to her babies.

"I think I made the decision in the beginning that that's what I wanted to do so I think I felt that I have to stick to it too."

Julie constantly compared herself with other mothers and felt she could prove herself as being a good mother, 'a better mum', by lasting longer at breastfeeding than other mothers did. Throughout her preterm breastfeeding experience, Julie was motivated by competing with other mothers. When asked why she persisted so long she replied:

" [to be] A better mum than anybody else. Certainly not a bad mum. I tried and I did what I could and, you know."

Julie's determination to continue and persevere with the expressing in order to increase the duration of lactation and at-breast feeding was important to her own sense of self as a mother. Her determination to continue breastfeeding was her marker of success as a good mother.

Fiona, on the other hand, by the time of her second interview, felt that she had expressed and breastfed for a sufficient duration to not be deemed a bad mother. At this stage, Fiona had been expressing for over eight weeks and was nearing the time of her baby's discharge home:

"It's, I guess, it's comforting to know that if I did give up now people wouldn't kind of go 'sigh' oh, you know, that's disgraceful, you know, it's your child, you should try harder, you know. I know that I have that support that people would understand and, so, but at the same time it was good to be, you know, you could just try a little bit harder."

At this stage of her breastfeeding experience Fiona was confident that her family and friends would support her decision to stop if she chose to. She felt she had passed the moral breastfeeding marker for duration, even though she had not yet achieved her goal of exclusive at-breast feeding.

Most of the women in this study continued to express their breast milk even though they had variable, and in most cases insufficient, milk volumes for the infants' daily needs. The routine of breast expression, and the strain this placed on daily life, was largely overlooked by these mothers because of the importance they placed on succeeding at this chosen part of care for their infant and for their own sense of achievement of motherhood. Breastfeeding was a highly valued undertaking in the preterm environment. It was important to these families that they were seen as persisting with their breastfeeding, at all costs. Sharon was the only mother who ceased her breast expressing efforts a few weeks after birth, and she did so after being unable to establish sufficient lactation to meet her baby's needs. She felt satisfaction for having given breastfeeding 'a try' and did not express feelings of moral failure as a mother. Sharon perceived her attempts to establish lactation were sufficient to be considered a 'good mother'; she had undertaken the moral imperative to breastfeed albeit for a short duration.

By believing that good mothers breastfeed their babies naturally and breast expressing was not the same as at-breast feeding, the women felt that some at-breast feeding was necessary to be a good mother. These mothers of preterm infants had significant innate pressure to succeed at expressing to enable them to do at-breast feeding. If they failed at expressing and never got to at-breast feed then, in their view, they never really got to breastfeed properly – they just provided breast milk. It is the at-breast feeds that are seen as important for the mother to achieve fully this aspect of her maternal role, as without these she has missed out on the essence of the breastfeeding experience she so desired.

### 4. 'So much is taken out of your hands'

All of the women in the study made some reference to the importance of 'my milk' to their motherhood, particularly in context of the preterm birth. The mothers felt that providing breast milk was the most active and valuable contribution they could make. Julie spoke of the importance of her milk:

"And it is good because I think you get that feeling that you're doing something for your babies, even though they're not with you. It's, you know, you just think babies and think milk is for babies and this is my milk and this is what you're contributing to your babies."

Similarly, Fiona said:

"there's a certain satisfaction in giving your child your milk ..."

This suggests that providing their own breast milk is one way that these mothers of preterm infants feel connected to their hospitalised infant. Lisa explained:

"With a preterm baby, I mean, it's just, there's so little you can do for them, you know, so much is taken out of your hands and it's just something [expressing breast milk], you know, even when I'm not with her, it's still something I know that I'm doing for her and I guess a lot of the time when I'm expressing at home I'm thinking about her, so, yeah."

For Lisa, there was so little mothering she felt able to do, as all the usual child care roles were performed by the hospital staff. Providing her breast milk was one mothering role she felt able to do and one that encouraged and enabled her to think about her baby. Even though she was separated from her milk and her baby, the breast milk acted as a form of physical and emotional connection for Lisa to her infant.

All of the mothers spoke positively of their breastfeeding role and the contributive value it offered them. Breast expressing for Sue was her major mothering task in those early weeks of her baby's life:

"Plus it's the only thing you can actually do for them because they are prem, apart from going and sitting and sort of touching them and you know changing their nappy occasionally and having them out for a hold I mean there's not really much you can do to care for them. But at least if you are giving ... expressing your breast milk, it is something that you know you feel you're contributing to their well being, I guess."

Nicole also spoke of the closeness she felt by providing her expressed breast milk for her baby:

*" [sigh] I feel closer to him than, yeah, I feel closer that he's having some of my milk, I'm giving him something to help him grow*."

Despite her desire to breastfeed, Fiona expressed the importance of the object breast milk for her daughter. This came at a time when her baby was too immature to breastfeed, and when the reality of at-breast feeding seemed unattainable, but the production of milk was real:

"You know, I've even considered maybe if she doesn't ever [at-breast feed], if we don't ever get it, if she just doesn't suckle, maybe I will just keep on expressing for months, you know, and just bottle feed her, so she's still taking my milk."

Lisa had similar thoughts about the need for the object milk as opposed to at-breast feeds:

"Well, I guess I will be really disappointed if I get to the stage of breastfeeding her and I can't, or something, she won't take it or, you know. But at least I'll know that, well, all this time she's had my milk anyway, so, and I've sort of thought about, well, if something happens and she doesn't latch on or she just, I can keep expressing and she can have it by bottle."

The disconnection of the milk from the mother became overwhelming in the experience of these women, even to the point that both women doubted ever achieving the 'real breastfeeding' that they desired. It is evident from these participants that there remained a connection of the baby receiving 'my milk', but, at the same time, it highlights the value of the object milk as being the important aspect of the preterm breastfeeding experience. Further discussion of the objectification of breast milk has been presented previously [27].

Providing breast milk for your child is an exclusive act of motherhood regardless of the infant's gestation. Expressing and providing breast milk was one way the participant mothers believed they were able to connect to their premature baby, despite their physical separation. Breastfeeding was one way these women were able to take part as a mother in the hospital nursery environment while most of their babies' needs were being attended to by the midwifery, nursing and medical staff. While expressed breast milk offered a form of connection and closeness for the participants to their newborn premature infant, it also became a burden [27,28,33]. Women received emotional satisfaction from providing breast milk for their infants and expressed great pleasure in being able to perform this motherhood role; however, it was evident that these women felt much moral obligation to persist with their breast expression. The mothers believed that not to achieve the desired breastfeeding, to have insufficient breast milk, or to give up would be a failure of their motherhood.

### 5. 'You have to do it' – Expressing is not a choice

In order for a mother to breastfeed a preterm infant weeks after birth, she must initiate and maintain her lactation. The only way of doing this is to 'wet nurse' another baby, or to express milk regularly from the breasts. 'Wet nursing' is not socially acceptable in mainstream contemporary Australian society and is not usually performed. Therefore, in our society, there is no option – expressing must be done. Expressing becomes a necessity for the mother who plans to breastfeed her preterm infant.

It was not by choice that participants were expressing, but rather a necessity by the choice of wanting to breastfeed. Expressing is the necessary precursor to the desired breastfeeding in their present situation. Helen spoke of the preterm breastfeeding experience as having two distinct parts. She says:

"It's like in two parts. There's the expressing part and all that and now there's this part [at-breast feeding], which is like the normal part. So it's got, like, a really nasty bit which is the expressing all the time, ... Yeah. It's horrible. It's alright, it's good, but it's, like, well, you're going to have to do it, but ... but it's really hard."

Helen refers to the milk production role as 'nasty' and 'horrible', but she is quick to downplay her feelings by suggesting it is 'alright' and even 'good' to be doing. By doing this she is reinforcing the good mother discourse. For Helen the task of expressing is not enjoyable and indeed creates a burden for these women, but the benefits are good; there are constant conflicting tensions. The tasks of breast expression create a burden for these women. For Helen, it is her continued lactation and ability to now at-breast feed that has made the milk producer role sustainable. The breast expression was horrible, nasty and good depending on which way it was considered; as everyday reality, as a role of motherhood or from altruism.

Breast expression is not unique to the preterm situation. For families with a term birth who choose to breastfeed, the infant will be feeding at the breast from birth and no expressing is usually necessary. However, families with an infant born at term may choose to express their breast milk if mother and child are to be separated for a period of time, such as for a social outing or if the mother goes back to work. In these circumstances, expressing is not a long-term necessity, but it is a choice to manage a short-term situation in addition to the usual established at-breast feeding. Expressing for a preterm infant is different to expressing for a term infant. With preterm birth, expressing is a necessity to establish and maintain breast milk supply, often for long periods of time, prior to the baby being discharged from hospital.

Julie found there was no choice of who does what with breastfeeding; if you want your babies to get breast milk then only the mother can do it; it was her gendered role.

"Well, of course you keep going. You do it because you feel you have to do it. You do it because you have to do it. That's where I suppose you feel a bit selfish there, because you're feeling you have to do it, because your husband can't do it."

The dependence on the woman as sole provider of milk was evident in the father's narratives [see also [33]]. The fathers showed an understanding of the role their wives/partners were required to undertake with regard to the expressing; however, some spoke of their regret at not being able to help or take over. Indeed, Peter thought breastfeeding was physically the mother's job, and therefore it was only she who suffered emotionally if it were not successful. He said:

"... it's only the mother that can do it, so it's only the mother that actually gets that feeling of incompleteness."

Despite the active role in assisting in breast-expressing tasks that some families participated in, such as milk transportation and pump setting up and cleaning, ultimately breastfeeding the preterm infant was a component of motherhood.

## Discussion

This paper has explored the breastfeeding experiences of parents of VLBW preterm infants. It has identified the connection between breastfeeding in this context and the mothers' sense of self and the maternal identity this gave them. The participant parents interpreted the importance of their lactation not only for the baby but also to their sense of motherhood. The meaning they ascribed to breastfeeding was far broader than simply that of infant nutrition. Benner and Wrubel explain that: 'In the phenomenological view the person is seen as attuned to and concerned with a world of significance' [[24], p. 97]. Breastfeeding was of great significance and meaning to the participant parents. It became the most important way mothers could actively participate in the care of their preterm child. In the NICU environment, supplying breast milk became a salient way in which to be a 'good parent'. Although with this connection came contradictions and ambiguities.

The participants demonstrated a strong relationship between providing expressed breast milk and which provided them with a sense of connectedness, despite the physical separation. Providing milk gave the participant mothers an important role in the care of their preterm infant. Providing expressed breast milk gave them agency as the mother. The mothers craved for an active parenting role, and this new role of breastfeeding mother was undertaken in a very positive and determined way. In the early days and weeks, mothers spoke of their breastfeeding as the only thing they could do for their baby. There is conflict in their conversations because they wanted to be closer to their baby and have a more participatory role, but were unable to do this at the time. Breastfeeding offered them a way in which to feel connected to their babies and feel a sense of satisfaction in assisting their babies' wellness; of giving 'my milk', whilst at the same time feeling the sadness of being separated. Providing expressed breast milk was filling a void in their maternal experience, albeit in only a small way. Similarly, Golembeski found that, for her cohort of 20 American mothers of high-risk infants, they 'had pride because this was milk that only they could provide' [[34], p. 44]. Moreover, '[o]ne mother said that bringing the milk to the hospital was "so exciting and it made me feel like a real mother"' [[34], p. 44]. Whilst there were only a few examples in Golembeski's study [34], it is evident that the findings of this present study are similar to those of other groups of study participants.

It is evident that the participant mothers were embroiled in a mass of conflicting ideas and notions. Expressing was viewed as a necessary step to achieve at-breast feeding although most women viewed it with conflicting emotions. They wanted to breastfeed so therefore they had to express their breasts; but expressing was not their choice. Furthermore, they wanted a participatory role in the child's care but could only provide breast milk; they were providing for their baby but not actually feeding them; someone else was administering their breast milk to their babies. The breast milk offered a means of connection to the baby, but only after disconnection of the mother and baby and the mother and her milk. They desired physical closeness and something special from their breastfeeding experience, but they were only able to have technical provision with mechanical equipment. They aspired to a shared parenting discourse, but expressing breast milk was only a mother's job. In addition, the outcome of her lactation rested solely with her. With so much conflict and tension surrounding their breastfeeding experiences, it is possible to see how it influenced both positively and negatively, their construction of motherhood.

For this group of Australian parents, the notions of 'good' motherhood and breastfeeding were synonymous. Although their breastfeeding was not a priority in the early days after birth, once the infant survived the first days of life breastfeeding was commenced. A delay in breastfeeding initiation for mothers of preterm infants has been documented before [35-38]; however, ascertaining qualitative reasons for this have been overlooked as evidence. This study found the onset of breastfeeding became significant for the participant mothers, as this gave them a symbol of mothering and assisted in the establishment of their new identity. The commencement of breastfeeding gave them hope that their baby would survive and eventually go home with them.

Many studies have explored the ways in which 'good mothers' and 'good fathers' have been defined – for example, by parents themselves, by experts and in texts on parenting [32], and such notions have changed little over the past 30 years [39]. Authors such as Chodorow [40] and Oakley [41] first questioned the way motherhood was defined by society. Since then, others have contributed to the debates about parenting and the social and cultural imperatives of good parenting [see for examples [16-18,42-46]]. These works show that a good mother is thought to be one who, for example, is self-sacrificial; always places the infant's needs above her own; is always present for the child; and displays unconditional love and care for the child while remaining calm, patient and in control of her own emotions. Therefore, the notions of 'good' motherhood are drawn from a child-centred discourse and have a strong emphasis on the importance of child welfare [39].

Marshall undertook an analysis of childcare and parenting manuals available to prospective and new parents [47]. The various accounts of motherhood in this literature construct motherhood as a wholly positive experience. Marshall coined the phrase the 'Ultimate Fulfilment' account to describe the way these 'manuals' direct women to expect motherhood to be satisfying and important: something special that is essentially a creative and positive experience [[47], p. 68]. Marshall goes on to explain that 'the Ultimate Fulfilment account constructs the experiences of the "natural" mother unproblematically, in essentially positive terms' [[47], p. 80], and, furthermore, 'shrouds any negative or ambivalent feelings a women may experience by characterising them as unnatural' [[47], p. 82]. The mothers in this present study worked hard to portray a positive tone in their experiences amid much conflict and tension. Undoubtedly, they had a positive expectation of at-breast feeding. It is evident that breastfeeding the preterm infant left them *desituated *from people, places, meanings, experiences and expectations – all involving new and unanticipated connotations, concerns and practices. To be *desituated*, is to have taken-for-granted aspects of life disrupted, and the new experience to be foreign [23]. These mothers worked hard to justify themselves as 'good mothers', ascribing to the ultimate fulfilment account, down playing negative and ambivalent feelings.

Hays [[17], pp. xii-xiii] also found that such 'manuals' consider mothers to be primarily responsible for raising children, a notion supported by Lupton [39] and Nippert-Eng [48]. Both mothers and fathers in this study have shown that they too believed that at least with regard to breastfeeding the newborn the mother is solely responsible. Hays argues that the contemporary cultural model of socially appropriate mothering takes the form of an ideology of 'intensive mothering' and that in this gendered model, mothers are expected to expend a tremendous amount of time, energy and money raising their children [[17], p. x]. Such images of motherhood and parenting are socially constructed and ever powerful [17].

Lupton and Fenwick, while discussing the perspectives of 'good motherhood' in the literature, recognise that such constructions of motherhood are based on mothers of healthy and well infants and that few sociologists have turned their attention to women whose infants are hospitalised immediately after birth [32]. Little discussion in the literature exists of how the notion of 'good' motherhood is assimilated into the practices of women whose experiences do not fit the normal schema of new motherhood, such as with preterm birth. Some researchers have begun to investigate and document such experiences [see for example [8,32,49-53]].

Susan Johnson, an Australian novelist and newspaper editor, suggests breast milk is deeply symbolic and 'to some women, at least, it represents nothing less than the whole of one's ability to mother' [[54], p. 52]. Recent research has shown that a vast majority of Australian women equate breastfeeding directly with the notion of being a 'good' mother and, in so doing, will maintain a strong commitment to breastfeeding despite enormous difficulties [8,13,19,39,55]. Breastfeeding is something women in those studies wanted to achieve. Furthermore, 'in the early months of first-time motherhood, breastfeeding was also a focal point of both intense satisfaction and intense distress' [[39], p. 56]. Lupton and Fenwick, while studying mothers of preterm infants, found that mothers placed more emphasis on breastfeeding as a component of 'good' mothering than did nurses [32]. They suggest this was primarily due to mothers seeing breastfeeding as a uniquely maternal practice, an act of love and nurture and one that was uniquely theirs that could not be overtaken by the nurses or anyone else [[32], p. 1019]. All participants in this present study maintained a strong commitment to the 'breast is best' rhetoric and directly equated their ability and persistence to provide expressed breast milk with their sense of self as being a 'good' mother. Their breastfeeding was considered an essential practice of good mothering given the preterm birth and the baby's 'need' for breast milk. Breastfeeding was portrayed as crucial to their maternal identity, as, in this environment, they were very limited in ways to perform their motherhood role.

## Limitations of this study

As with all research, there are limitations with this work. This study was conducted on a relatively small sample group of 17 parents; 10 mothers and 7 fathers. This study was conducted with Australian middle-class Caucasian families who chose to breastfeed their preterm infants. However, the clinical management of preterm infants and the care provided to breastfeeding mothers of preterm infants is not the same in all institutions and therefore the parental experience of breastfeeding preterm infants will not necessarily be the same. As human beings we all have different views, opinions, history, expectations and experiences, and no two will be exactly the same. However, commonalities do exist in similar, or like groups experiencing similar human experiences such as breastfeeding preterm infants. Consequently, the thoughts, feelings and practices described throughout this study will not be generalisable to all parents of preterm infant populations, but it is hoped that the commonalities and highlighted differences with the provided interpretation have revealed meanings of the human experience.

## Conclusion

The findings of this study have implications for all who care for parents of preterm infants as well as breastfeeding advocates. The results presented here, extend the body of work that has identified the powerful discourses that currently position breastfeeding as crucial to the construction of motherhood. The participant mothers highlighted the positive aspects of their breastfeeding as it allowed them to connect to their absent babies and to perform as mothers when all other mothering tasks were taken out of their hands. This ability to provide breast milk made them feel valuable and unique – being the only person able to provide this milk. Therefore providing breast milk was very important for their sense of motherhood. Despite the sense of connection afforded, the period of breast milk provision was also experienced by these mothers as disempowering, disconnected and negative [28]. It is evident that the association of breastfeeding success with mothering success only jeopardises some families' self-esteem and sense of parenting ability. In Australia, the promotional messages about breastfeeding being best have been very successful in improving breastfeeding rates. However, Australian parents also associate breastfeeding with good parenthood and crucial to the mothering role. This view places undue stress and anxiety on all women and even more so on women whose experiences do not meet the norm, such as parents of preterm infants. These findings suggest it would be beneficial to find complementary ways to connect preterm infants and their parents in the preterm nursery environment, and find more realistic and sustainable ways to promote and support breastfeeding. As a health professional, I recognise this poses new challenges of how we might promote and protect breastfeeding for preterm infants while simultaneously giving families a realistic picture of the difficulties and potential outcomes of breastfeeding.

## Competing interests

The author declares that she has no competing interests.
